# Cellular Response to ELF-MF and Heat: Evidence for a Common Involvement of Heat Shock Proteins?

**DOI:** 10.3389/fpubh.2017.00280

**Published:** 2017-10-18

**Authors:** Olga Zeni, Myrtill Simkó, Maria Rosaria Scarfi, Mats-Olof Mattsson

**Affiliations:** ^1^Institute for Electromagnetic Sensing of the Environment (IREA), National Research Council, Naples, Italy; ^2^SciProof International AB, Östersund, Sweden; ^3^AIT Austrian Institute of Technology, Center for Energy, Environmental Resources and Technologies, Tulln, Austria

**Keywords:** extremely low frequency magnetic fields, heat, combined exposures, heat shock proteins, thermotolerance

## Abstract

It has been shown that magnetic fields in the extremely low frequency range (ELF-MF) can act as a stressor in various *in vivo* or *in vitro* systems, at flux density levels below those inducing excitation of nerve and muscle cells, which are setting the limits used by most generally accepted exposure guidelines, such as the ones published by the International Commission on Non-Ionizing Radiation Protection. In response to a variety of physiological and environmental factors, including heat, cells activate an ancient signaling pathway leading to the transient expression of heat shock proteins (HSPs), which exhibit sophisticated protection mechanisms. A number of studies suggest that also ELF-MF exposure can activate the cellular stress response and cause increased HSPs expression, both on the mRNA and the protein levels. In this review, we provide some of the presently available data on cellular responses, especially regarding HSP expression, due to single and combined exposure to ELF-MF and heat, with the aim to compare the induced effects and to detect possible common modes of action. Some evidence suggest that MF and heat can act as costressors inducing a kind of thermotolerance in cell cultures and in organisms. The MF exposure might produce a potentiated or synergistic biological response such as an increase in HSPs expression, in combination with a well-defined stress, and in turn exert beneficial effects during certain circumstances.

## Introduction

Cellular stress can be caused by a number of physical and/or chemical factors. These include non-physiological temperatures (heat, cold), oxygen deficits, acid–base imbalances, toxic compounds, ionizing radiation, etc. (1–3). Heat stress has been extensively investigated since decades. If the temperature is high enough, cells are responding with a heat stress response (discussed in further detail below) aiming to protect the cell from undergoing apoptosis and/or necrosis [see, e.g., Ref. (4–6) for comprehensive reviews]. A number of cellular processes are affected, leading to the survival of the cell. Prior to this event, the cell is increasing the expression of genes encoding the so-called heat shock proteins (HSP) (7), which execute the necessary functional adjustments. Interestingly, if a so-called mild heat stress (39–43°C) at a level below a lethal temperature (44–45°C) occurs, the cellular response is somewhat different. This is reflected in both the gene expression pattern (8, 9) and the physiological responses within the cell (10). In addition, there is evidence that pretreatment with a mild heat stress significantly improves the resilience of the cell if it is later exposed to a high (lethal), temperature (11–13). Repeated mild heat stress has even been shown to stimulate proliferation and differentiation in human adipose tissue-derived mesenchymal stem cells (14). There are some intracellular “temperature sensors” existing, such as damaged (miss-folded or aggregated) proteins that are appearing due to high temperatures. Regarding mild heat stress, the plasma membrane and also intracellular membranes have been suggested to initially respond to the increased temperature and subsequently propagate the signals leading to various downstream activities (15).

It has been repeatedly shown that magnetic fields in the (extremely) low frequency range (ELF MF) can act as a stressor in various *in vivo* or *in vitro* systems. Such effects have been documented also at flux density levels that are below those causing direct stimulation of nerve and muscle tissue and the induction of retinal phosphenes. Protection against these effects is the rationale behind the recommended exposure limits found in most generally accepted exposure guidelines, such as the ones published by ICNIRP (16). As an example, for power frequency magnetic fields (50 or 60 Hz), the ICNIRP reference level for general public exposure is 0.2 mT.

The direct cellular targets in producing these effects are not known, and there is no generally accepted interaction mechanism that can explain such “low level” effects [see, e.g., the last Opinion from the Scientific Committee on Emerging and Newly Identified Health Risks for a recent review of health-related effects of EMF exposure and a discussion about mechanisms (17)]. It has been demonstrated that ELF-MFs can activate the cellular stress response by inducing the expression of stress response genes, e.g., *HSP70*, and increased levels of stress proteins, such as HSP70. Some authors highlighted that the onset of stress response by MF exposure should be considered as an indicator of the harmful potential of ELF-MF, based on the concept that stress response is defined as a defense reaction of the cell to damaging agents (18, 19). Other authors have suggested that beneficial effects (such as protection against various stressors) can be induced by stress, including also ELF-MF if they act as a mild stressor (20–22).

The MF exposure is considered as a weak cellular stressor since detected MF effects are very modest. Thus, the MF interaction with biological systems is difficult to study since cellular homeostatic mechanisms may quickly compensate for the physiological disturbances.

Besides the studies reporting on the heat shock response evoked by ELF-MF exposure alone, some investigations have been carried out in which the effects of ELF-EMF have been addressed under mild thermal stress conditions. Here, we provide an overview of some of the presently available data on cellular responses, especially regarding HSP expression, due to single and combined exposure to ELF-MF and heat, with the aim to compare the induced effects and to detect possible common modes of action.

## Chaperones—Heat Shock Proteins

HSPs are belonging to the most conserved of all proteins in prokaryotes and eukaryotes. Some of the HSPs expression can be induced by a variety of physiological and environmental factors, such as heat, oxidative stress, or anticancer drugs [for recent reviews see Ref. (23)]. HSPs act as molecular chaperones. These proteins play an essential role in refolding stress-induced miss-folded proteins to prevent protein aggregation and thus to tolerate and “repair” certain lethal conditions and restore the protein homeostasis. Thus, intracellular HSPs have a cell-protective function. HSP are classified into five groups according to their molecular size: HSP100, HSP90, HSP70, HSP60, and the small HSPs (23). While HSP90 is constitutively expressed, HSP70 and HSP27 are not or to very low degrees expressed in non-stressed cells. HSP70 and HSP27 are highly inducible by different kinds of stresses and can be targeted to different cellular compartments. Once they are induced, these proteins can modulate many different cellular functions to protect the cell or to be involved in the apoptotic-signaling pathway.

Mild (heat) stress enhances chaperone action in protein maintenance and detection of protein damage by activating specific signaling pathways. This leads to increased recruitment of HSPs, which decreases the burden of aberrant proteins by allowing proteins to refold to their native conformation. The heat shock response requires a specific transcription factor, namely the heat shock factor HSF1 (24). When activated, it is binding to the heat shock element (HSE) on the DNA, which initiates the assembly of the transcription machinery. Under non-stressed conditions, inactive HSF1 remains bound to the HSPs in the cytoplasm and is thus repressed. Stress (heat shock) can trigger the HSF1 dissociation from HSP and the release of HSF1 subsequently leads to nuclear translocation. In the nucleus, HSF1 binds to HSEs where the transcription of *HSP* genes starts (24). It has also been shown that cells derived from mice lacking HSF1 are sensitive to stress and are unable to develop thermotolerance or to induce heat responsive genes upon heat shock [summarized in Ref. (25)].

HSP70s are powerful anti-apoptotic proteins and block apoptosis at a number of different levels (26, 27). It has to be pointed out that the basal level of HSPs is strongly cell and tissue dependent. It has been shown that certain cancer cells “rely” on the HSP70 for survival, with high basal level of the protein. This provides these cells with antiapoptotic mechanisms, which enhance cell growth and suppress senescence, and protect against cytostatic drugs and radiation therapy (28). However, while intracellular HSP70s play a key role in proteomic homeostasis, extracellular or membrane bound HSPs mediate immunological functions, and stimulate innate and adaptive immunity [for review see Ref. (29)]. Thus, HSPs have a dual role: intracellular cell protective and extracellular cell signaling for immune modulation.

## ELF-MF and Heat Shock Proteins

In EMF research, HSPs have often been considered as “hazard” marker proteins. Many studies investigated the induction of HSPs also on the gene and protein expression levels using different exposure conditions, but with inconsistent outcomes. Considering that the basal expression levels of HSPs are cell type dependent, we here analyze the responses according to the cell type. The list of identified studies is presented in Table S1 in Supplementary Material.

In some studies, no HSP-related effects were detected after ELF-MF exposure ranging from a few μT to mT and from minutes to 24 h, using different cell types such as astroglial cells (30), HL-60, H9c2, and Girardi heart cells (31, 32), and human keratinocytes (33). Also in human leukocytes no changes on the gene expression level (mRNA) was detected (34). However, an interesting study investigated the expression of the luciferase gene contained in a plasmid labeled as electromagnetic field-plasmid, which is the human HSP70 promoter isolated from whole blood upstream of the luciferase gene. The plasmid vector was transfected into two different cell lines (INER-37 and RMA E7) that were later exposed to MF. An increased luciferase gene expression was observed in INER-37 cells exposed to MF compared to controls, but MF exposure had no effect in the RMA E7 cell line (35), suggesting a cell type-dependent susceptibility to MF exposure.

In contrast to the absence of effects in the studies above, exposure has also caused changes in HSP levels in a number of primary or non-transformed (“primary like”) cell lines. The effects were seen as an induction of *HSP* genes on both the mRNA and protein levels in various cell types such as human primary osteoarthritic chondrocytes (36), mouse macrophages (37), transfected rat primary fibroblasts (RAT1 cells) (38), transfected rat primary cells (39) or cardiomyocytes isolated from neonatal Sprague-Dawley rats (40), porcine aortic endothelial cells (41), endothelial cells (SPAE, HUVECs), and human fibroblasts (HuDe, WI-38) (42). Considering that the basal level of HSPs expression is strongly cell type dependent, it can be hypothesized that primary cells or primary-like cell lines have a lower basal HSPs level, and thus that these cells are rather easier inducing HSPs than cancer cell lines. However, increased levels of both HSP27 and HSP70 were detected in a number of cancers (breast cancer, ovarian cancer, osteosarcoma, endometrial cancer, leukemias and renal cell tumors) relative to the levels in non-transformed cells [for a review see Ref. (43)].

MF-induced increases in HSP expression levels were also detected in a number of immune relevant cell lines: macrophages [RAW264 cell line (44)], human leukemia and lymphoma cells [CEM, HL-60, U937, K562 cells, THP-1 cells (20, 42, 45–47)]. Immune relevant cells or cell lines furthermore produce extracellular HSPs as a signal molecule for immune response [e.g., Ref. (48)]. So far, no investigation has been focusing on extracellular HSPs expression/release after ELF-MF exposure.

The hypothesis that non-stressed cells or organisms are quite responsive to HSP induction after ELF-MF exposure is strengthened by some *in vivo* studies in invertebrates: the expression of HSP70 was elevated after ELF-MF exposure in the bivalve *Mytilus galloprovincialis* (49), in the planarian *Dugesia dorotocethala* (50), and also in *Dictyostelium discoideum* cells (51). Using the bacterium *Escherichia coli*, significantly higher level of DnaK and GroE (corresponding to eukaryotic HSP) proteins as well as changes in HSP-related protein synthesis were detected (52). However, Nakasono et al. detected no effects in *E. coli* (53).

## Combined Exposure to ELF-MF and Heat

### ELF-MF Exposure Potentiates the Effects of Heat on HSP Induction

A summary of the identified eight studies is presented in Table 1. In six of eight articles, the potentiating effect of ELF-MF on thermal stress in both cell models and organisms has been demonstrated using *HSP* mRNA levels/synthesis as the outcome. Different cell models, from normal to cancer tissues were employed, and subjected to different exposure conditions such as different magnetic flux densities, exposure durations, and coexposure protocols. Mild and severe heat stress conditions (40–44°C) have been employed for the coexposure protocols, which varied depending on the cell type used in the experiments.

**Table 1:** Combined exposure to ELF magnetic field and heat: studies reporting on a potentiation of heat-induced effects.

Reference	Model system	Exposure conditions	Thermal stress conditions	End point (s)	Outcome of coexposure	Comments
(54)	Human adenocarcinoma cells (HeLa)	60 Hz	43°C	Luciferase gene expression in cells transfected with HSP70 promoter	Synergistic effect (luciferase gene expression)	Increased luciferase expression after MF alone
		80 µT	20 min			
		20 min	Concurrent			

(42)	Endothelial cells from pig pulmonary arteries (SPAE)	50 Hz	44°C	mRNA level and synthesis of *HSP*70	Enhanced accumulation and translation of the heat-induced *HSP*70 mRNA	MF induced an increased level of inducible *HSP*70 in endothelial, lymphoma, and leukemia cells. No effect in human fibroblasts
		680 µT	30 min			
		24 h	Postexposure			

(47)	Human myeloid leukemia HL-60 cells	50 Hz	43°C	mRNA level and synthesis of *HSP*70A/B/C; *HSC*70; *HSP*27, 60, 75, and 90 (α and β)	Strong induction in *HSP*70 gene family in the interval 40–80 µT	Increased *HSP*70 mRNA levels and synthesis after MF alone
		10–140 µT	30 min			
		30 min	Concurrent			

(55)	Breast MCF 7 and cervical HeLa (cancer); breast epithelium HBL-100 (healthy)	50 Hz train pulsed MF (2 s burst and 1 s rest)	40°C	HSP70 synthesis, cell proliferation	Increased HSP70 by MF under thermal stress in both healthy and cancer cells	No effects of MF alone
			42°C			
			Concurrent			
		34 mT				
		0–12 h				

(57)	*Drosophila* wild-type CS and mutant w1118 flies	50 Hz	35°C	Death rate, life span, locomotion, *HSP* 22/26/70 mRNA levels, oxidative stress	MF enhanced thermal stress effects	Some gender differences, sporadic MF effect at 25°C
		3 mT	Concurrent			
		2 and 24 h				

(56)	Transgenic *C. elegans*	50 Hz	28 and 29°C	Expression of *HSP*16 and 70 promoters upstream of lacZ at 100 µT	EMF enhanced the lacZ reporter gene expression under the control of *HSP*16 or 70 promoters	No effect of MF alone
		50, 100 µT	Concurrent			
		1 h				

(58)	Wistar rats	60 Hz	43°C for 12 min before MF	Histopathological and histomorphometrical analysis of rat testes	MF aggravates the effects of thermal stress	
		1 mT				
		15, 30, 60 days				

(59)	*Drosophila melanogaster*	50 Hz	34–37°C	Embryogenesis anomalies	MF aggravates the anomalies due to thermal stress	
		100 µT	Concurrent			
		30 min				

Interestingly, when HeLa and HL-60 cancer cells were subjected to comparable magnetic flux densities (10–140 µT), exposure durations (20–30 min) and concurrently heat stressed at 43°C, a stronger HSP70 expression was attained in coexposed cells compared to the thermally stressed ones. Also the MF exposures alone showed increased HSP70 expression levels (47, 54). When both normal (breast epithelium HBL-100 and endothelial cells from pig pulmonary arteries SPAE) and cancer cells (MCF-7 breast cancer and HeLa cervix carcinoma cells) were subjected to higher flux densities (680 µT and 34 mT) for longer exposure times (0–24 h), and were heat stressed in the range 40–44°C both concurrently and post MF exposure, again increased *HSP70* mRNA levels/synthesis was seen in the coexposed samples, compared to thermally stressed samples. Interestingly, under such conditions MF exposure alone did not exert any effect. A comparative study regarding HSP70 expression in normal and malignant cells was performed by Tsurita et al. (55). The authors concluded that the applied exposure potentiates the effect of thermal stress in both normal and malignant cells. However, they also pointed out that malignant cells derived from different organs respond differently to EMF exposure.

The increase in *HSP70* mRNA levels after heat and MF exposure was detected also in animal models, such as in transgenic *Caenorhabditis elegans* nematodes and in *Drosophila malanogaster*. In *C. elegans*, 1 h concurrent exposure to 50 and 100 µT under 28 and 29°C thermal stress was carried out (56) while, in *Drosophila*, 2 and 24 h concurrent exposures were carried out at 35°C (57). The potentiation of heat-induced HSP16 (56) and HSP22 and HSP26 (57) expression by concurrent MF was detected.

The potentiation of the thermal stress effects was also gained in two additional articles, not investigating HSP induction. In these studies, histophathological and histomorphometrical analysis of testes in Wistar rats and embryogenetic anomalies in *Drosophila melanogaster* were performed. In the first study, rats were stressed at 43°C for 12 min before 60 Hz MF exposure for 15, 30, and 60 days at 1 mT magnetic flux density (58), whereas in the *Drosophila* study, samples were concurrently heat stressed (34–37°C) and exposed to 50 Hz, 100 µT for 30 min (59).

### ELF-MF Exposure Protects from Secondary Effects of Heat

Here we consider as secondary the effects that appear after a certain time of the exposure, as a down-stream effect: examples are modulation of cell proliferation and apoptosis. The protective effect of MFs after thermal stress has been investigated in four studies (Table 2) in both cell models and organisms. Thus, human myeloid leukemia HL-60 cells, and HL-60 with a mutated retinoic acid receptor-α gene (*HL60-R*) have been used in the studies, as they are heat sensitive cell types. Human Burkitt lymphoma (Raji) cells were also employed in one of the studies, as they are heat insensitive cells. Different exposure conditions in terms of magnetic flux density, exposure duration, and coexposure protocols were applied. Cell survival, cell cycle progression and apoptosis have been analyzed, and in some cases also *HSP* mRNA levels have been evaluated. Specifically, HL-60 cells concurrently exposed for 30 min to 50 Hz MF (60 µT) and thermal stress, resulted in protection from the cell growth arrest that is normally induced by 41°C (47, 60). Moreover, a consistent induction of *HSP70* mRNA levels above the level of each stressor alone, was also detected after coexposures (60). In another study, heat sensitive HL-60 and HL-60-R cells, as well as the heat insensitive Raji cells, pre-exposed for 12 h to 60 Hz MF (150 µT) were protected from thermal stress effects (1 h at 43°C) such as induced apoptosis, with the protection lasting up to 48 h post exposure (61). The protective effect detected in cell models was also demonstrated in fertilized eggs of the dipteran *Sciara coprophila* preconditioned for 30 min with ELF field (60 Hz, 8 µT) at 20°C, and then treated at 36.5°C for 60 min (lethal temperature). Eggs had on average an 82% increase in survival and a 114% increase in HSP70 synthesis. The authors also demonstrated that thermal preconditioning at 32°C was not nearly as effective, inducing a 44% increase in eggs survival (22).

**Table 2:** Combined exposure to ELF magnetic field and heat: studies reporting protection from secondary effects of heat.

Reference	Model system	Exposure conditions	Thermal stress conditions	End point (s)	Outcome of coexposure	Comments
(60)	Human myeloid leukemia HL-60 cells	50 Hz	37, 39, and 41°C	Cell survival, cell cycle analyzed up to 10 days postexposure	MF protects from the cell growth arrest induced by 41°C	Authors hypothesized specific interactions of HSP70 with molecules regulating cell cycle, apoptosis
		60 µT				
		30 min	Concurrent			

(61)	Human myeloid leukemia HL-60 cells	50 Hz	41°C	*HSP* mRNA levels (27, 60, 70, 75, 78, 90), cell cycle progression	Strong induction of *HSP*70 above the level of each stressor.	*HSP*70 induced by MF alone.
		60 µT	Concurrent			
		30 min				*HSP*27, 75, and 78 induced after only thermal stress
					Negation of the thermal stress-induced cell cycle arrest	

(62)	Heat sensitive (HL-60 and HL-60R) and heat-insensitive (human Burkitt lymphoma, Raji) cells	60 Hz	43°C	Apoptosis	12 h MF protects from heat-induced apoptosis in all the cell types	The protective effect lasts up to 48 h postexposure
		150 µT	1 h			
		4, 12, 24 h	Postexposure			

(22)	Fertilized eggs of *Sciara coprofila*	60 Hz	36.5°C (lethal temperature)	HSP70 synthesis, survival	114% increase in HSP70 levels, 82% increase in survival	Preconditioning at 32°C was not so effective in increasing survival
		8 µT				
		30 min	60 min			
			Post exposure			

## Summary and Concluding Remarks

Cells respond to a variety of environmental and physiological stresses by rapidly synthesizing stress response proteins such as HSPs. Induction of the HSPs protects cells against the harmful consequences of a diverse array of stresses, including those imposed by thermal stress (62). A number of studies suggest that also ELF-MF exposure can cause increased HSP expression, both on the mRNA and the protein levels. Furthermore, experiments using combined exposures to ELF-MF and heat indicate that the MF potentiates the effects of heat on HSP expression. As a consequence, the damaging effects of heat exposure alone are counteracted by the MF exposure.

Not all studies employing MF exposure show increased levels of HSP transcripts or peptides. A wide range of flux densities were used in the different experiments, ranging from tens of μT up to tens of mT (see Table S1 in Supplementary Material). No clear dose–response pattern appears from the studies, and there is no study that shows a threshold for any of the observed effects. Furthermore, it seems that short exposure duration and very low magnetic flux densities are sufficient for obtaining a response. These results are consistent with the heat shock response which is a rapid and transient gene-expression program (3).

The differential sensitivity to MF cannot be ascribed to specific exposure conditions, according to the available studies. More likely, the status of the biological material may play an important role for any HSP induction. In general, research on biological effects of EMF suffers from inconsistencies in outcomes regarding various end points. This problem was addressed in detail by McCreary et al. (63) who analyzed real-time cytosolic Ca^2+^ fluctuations after ELF-MF exposures in Jurkat E6.1 cells. They could consistently detect effects if the analysis considered various covariates including pH, cell cycle stage, and response to a Ca^2+^ agonist. The authors stressed that the MF effects may be difficult to detect unless specific consideration is made regarding the biological material’s characteristics. This has also been pointed out by other authors [*inter alia* (64–66)] who all made a point of listing specific criteria that were needed to be fulfilled, on the single cell level, in order to detect a MF response.

Another possible factor regarding inconsistent results may refer to the cell type (e.g., tissue origin, concert of receptors, and metabolic state), which has been used since similar cell lines respond in some cases and do not in others. The lack of effects might also be due to differences in experimental conditions used (e.g., type of serum). Neoplastic transformation may be still another relevant factor to consider. In summary, the presented studies do not provide any support for either that sensitivity is restricted to any specific cell type or to transformed or normal cells. Of the included studies, a direct comparison among cancer cells and healthy cells was carried out under similar experimental conditions, and similar cellular responses were obtained in both cell types (55). The investigations reviewed herein did not provide evidence for sensitivity of one cell type with respect to any other.

A possible direct evidence of a common involvement of HSPs in cellular response to MF exposure and heat treatment can be found in the work by the Goodman group. They reported that HL-60 cells exposed to MF to 8 µT for 20 min showed a strong HSF1 activation with its subsequent binding to an HSE sequence located upstream of the classical heat-shock domain. The authors suggested that this electromagnetic responsive element domain lies between −230 and −160 on the HSP70 promoter and also contains sites of binding for the MYC protein (67, 68). The studies also showed increased MYC levels after exposure, and the authors ascribed this effect to an MF-mediated HSP activation. However, other studies from independent laboratories could not confirm the findings regarding MYC (32, 33).

Along this line, by using endothelial cells, Alfieri et al. detected a weak transient activation (ca. fourfold) of HSF1 after MF exposure to a 300–680 µT, although without changes in the *HSP70* mRNA level or the synthesis of HSP70 (42). Moreover, the authors also demonstrate an accumulation of the inducible HSP70 protein, which they ascribed to a reduced degradation of the protein. On the other end, severe heat shock (44°C) induced a strong activation of HSF1-HSE binding (ca. 13-fold) (42). An accumulation of HSP70 due to reduced degradation was shown in porcine aortic endothelial cells as well (41). Based on these data, it is unclear whether the accumulation or the induction of HSP70 is causing an increased protein level. However, in a recent finding it was shown that HSF1 can also be directly regulated by MEK *via* phosphorylation, as part of the MAPK/ERK cascade, and not only by stress-induced signaling (69). The MEK-HSF1 regulation mediates the cell-environment interactions, beyond others, and critically regulates heat shock response *via* the RAS-MEK-ERK signaling. Unfortunately, there are no studies showing any involvement of the MEK-HSF1 regulation after ELF-MF exposure.

Experiments investigating coexposures of heat and MF provide support for the notion that the MF potentiates the effects of heat. In the majority of the articles, we have analyzed, and in particular in all the experiments carried out on cell models, a potentiation was demonstrated in terms of HSPs: the MF exposure increased the heat-induced HSPs expression levels. The design of the experiments does not allow any interpretation of whether the two agents employ the same pathways to achieve increased HSP expression. The experiments were furthermore done with MF exposure both before and at the same time as heat stress. Thus, the exposure regimes are not providing any consistency.

An interesting finding is that MF exposure provides protection against heat-induced effects such as apoptosis, cell cycle disturbances, or proliferation inhibition in both cell models and in organisms. These protective effects were achieved when the MF exposure was applied before, or concurrent with heat. Tokalov et al. found that heat-induced cell cycle arrest was counteracted by MF exposure, which took place at the same time as the heat treatment. This condition also produced significantly higher levels of HSP70 than either MF or heat alone (60). A protection against heat-induced lethal effects was also documented in a study on fertilized *Sciara* eggs (22) where increased survival rate was observed, that was accompanied by an increased level of HSP70. These findings are in agreement with the suggested cytoprotective effects and regulatory roles of HSPs on apoptosis and the cell cycle (70). It is thus possible that those studies that show protective effects of MF exposure against heat stress, but did not investigate HSPs, could demonstrate a similar increase in HSP levels.

In only one article, a suppression of the HSP70 levels induced by heat was attained, and no significant protection from secondary effects was found. In this study, by Miyakoshi et al., concurrent exposure of HL-60-RG cells to 60 Hz (50 mT) for 5–20 h lowered the 40 and 42°C-induced HSP70 synthesis, while the cytotoxicity of 42°C was not affected. Such a suppression of HSP70 synthesis was not induced by 0.5 and 5 mT MF. No effect of MF given alone or before or after thermal stress was attained (71). These findings are particularly interesting since they strengthen the idea that coexposure conditions resulting in an increase in heat-induced HSPs only are effective in inducing protection from secondary effects of heat.

It is worthwhile to note that in a study employing human peripheral blood cultures exposed for 4 h to 50 Hz MF in the range 0–100 µT no effect was elicited on the expression of genes encoding *HSP27, HSP70A* and *HSP70B*, at any of the magnetic flux densities. Moreover, the gene expression in cells exposed to MF at 40°C was not changed compared to cells incubated at 40°C without field exposure (34).

Another study has been recognized, in which MF exposure did not modify the effects of heat, where the microvesicle velocity was monitored to study the cellular stress level. Primary rat astrocytes were treated at 45°C for 10 min before 50 Hz MF exposure for 1 h at 100 µT. Increase in the microvesicle velocity was detected after both MF exposure and heat treatment alone, which was not modified by the combined treatments (72).

It has been described by Park et al. that the major difference between the cellular responses to mild and severe heat stress is the adaptation to growth conditions, meaning that during mild heat stress (39–43°C, depending on the cell type) cells are showing an increased cell proliferation, whereas at severe heat stress (44–45°C) cell cycle arrest and apoptosis are induced. Also, differences in the activity of certain signaling pathways were identified. Thus, mild heat stress causes activation of multiple Ras signal pathways (including the ERK1/2), the phosphatidylinositol-3 kinase-Akt/protein kinase B (PKB)-glycogen synthase kinase-3b pathway, and Rho-Rac1-NADPH oxidase pathways. These pathways positively regulate cell cycle progression and differentiation, but are not active after severe heat stress (10).

Some available studies provide hints that MF also acts like a mild stressor. For example, after MF exposure (100 µT, 50 Hz) an induced transient activation of p38, JNK and ERK1/2 was shown in NB69 cells accompanied by changes in the cell cycle progression (73). The authors showed that the MF-induced proliferative effects were exerted through sequential upregulation of MAPK-p38 and ERK1/2 activation and that the upregulation likely was mediated by a ROS-dependent activation of p38. In addition, Frahm et al. showed that MF exposure (50 Hz, 1 mT) caused slight and transient decreases in levels of clathrin, adaptin, PI3-kinase, PKB, and PP2A after short-term exposures (2 h or less) (37).

Interestingly, there is evidence of cross-talk between oxidative stress and other stress-responding pathways. For example, oxidative stress is known to cause an increase in the expression of certain inducible HSPs, particularly HSP27. HSPs have been reported to protect against many stresses apart from heat shock, including heavy metals, radiation, nitric oxide, and other oxidants (25). It is known that different cell types react differently to (mild) stressors that could be depending on the different cell functions. Thus, cells in which HSP70 plays an important role in regulating, e.g., cell cycle or apoptosis might respond on a different way to oxidative stress compared to cells where the function of HSP70 is mainly to regulate protein folding. A direct effect on the oxidative stress-HSP70 axis was recently shown by Mannerling et al., who employed human erythroleukaemia K562 cells for their experiments (45). After 50 Hz exposures (1 h; 0.025–0.10 mT), both HSP70 levels and ROS were elevated compared to both sham controls and heat treatment. By using radical scavengers, it was found that MF-induction of HSP70 was inhibited, supporting that MF initially triggers radical formation which leads to increased levels of HSP. This study can thus be interpreted so that both mild heat stress and ELF-MF stimulate HSP expression, but *via* different pathways. The authors did unfortunately not investigate effects of coexposures to heat and MF. A recent study showed very similar results under comparable experimental conditions, with the exception that RAW mouse macrophages instead of human K562 cells were investigated (44).

In summary, on the basis of the available data dealing with single exposure to ELF-MF showing HSP expression modulations, no (co)relation to MF-dose, specific exposure conditions, or cell type could be identified. The data regarding coexposures to MF and heat are very similar, and we cannot derive any consistent clue regarding a possible common mode of action. There is some evidence that MF and heat might act as costressors inducing thermotolerance in cell cultures and in organisms. The MF exposure might produce a potentiated biological response, such as the increase in HSPs expression in combination with a well-defined stress, and in turn exerts beneficial effects. It is also possible that ELF-MF exposure protects the cells *via* desensitization against heat stress, and so from secondary effects. Since the mode of action is not clear, we can only speculate if the applied temperature or the MF parameters or the cell type used (cell receptors and metabolic state, culture media, serum, etc.) is a relevant factor influencing the outcome, or if all together are important players in the biological response. Since systematic investigations are not available, we have to consider that beside the physical parameters used, more knowledge is needed about metabolic status and the absolute basal HSP levels of the cell models. Experiments, carried out under strictly controlled conditions from both electromagnetic and biological point of view, are needed to address specifically the underlying mechanisms involving HSPs and cellular responses to ELF-MF and heat.

## Author Contributions

OZ, MRS, MM, and MS: design the work; acquisition, analysis, or interpretation of data; drafting or revision of the work; final approval of the version to be published; agreement to be accountable for all aspects of the work in ensuring that questions related to the accuracy or integrity of any part of the work are appropriately investigated and resolved.

## Conflict of Interest Statement

The authors declare that the research was conducted in the absence of any commercial or financial relationships that could be construed as a potential conflict of interest.
