# Supplementary material for: Cellular Response to ELF-MF and Heat: Evidence for a Common Involvement of Heat Shock Proteins?
Source: Front Public Health. 2017 Oct 18;5:280. doi: 10.3389/fpubh.2017.00280 (PMC5651525; doi:10.3389/fpubh.2017.00280)
Supplement: Supplementary file 1 [file table_1.docx]

Table 1 Supplementary Studies reporting on ELF magnetic field exposure and heat shock protein effects

| **Reference** | **Exposure conditions** | **Model systems** | **Outcome** |
| --- | --- | --- | --- |
| [44] | 50-Hz  0.1 or 0.5-mT  1 h, 17 h or 24 h | RAW264 macrophage cell line | Increase in O_2_- production and the expression of HSP70 protein. |
| [20] | 50 Hz  1 mT  4-6 h | THP-1 cells (human monocytic leukemia cell line) induced with S. Aureus or interferon gamma/lipopolysaccharide (ifγ/LPS). | ELF-EMF alone caused a time-dependent increase of HSP70 levels. When cells were induced with S. Aureus or ifγ/LPS, field application produced higher levels of HSP70. ELF-EMF suppressed caspase-9 activation by a small extent. |
| [42] | 50 Hz  680 µT  24 h | Endothelial (SPAE, HUVEC), human leukemia and lymphoma cells (CEM, HL-60, U937), and human fibroblasts (HUDE, WI-38) | Increased level of HSP70 in SPAE, HUVEC, HL-60, U-937 and CEM cells.  Fibroblasts not modified. |
|  |  |  |  |
| [51] | 50-Hz  300-μT  24 h | *Dictyostelium discoideum* cells. | Increase in HSP70 that returned to the control value after a 24h of exposure. |
| [41] | 50 Hz  1.0 mT rms  4 h | Porcine aortic endothelial cells (PAEC) | Increase in mRNA levels of *HSP*27, *HSP*70, and *HSP*90 but not on the protein levels, HSP27 was partially delocalized to the nucleus. |
| [30] | Static or 50 Hz  1-mT  1h | Astroglial cells | No effect on HSP25, HSP60, and HSP70 protein levels. |
| [36] | 100 Hz, with musically modulated EMFs  30 min/day for two weeks | Human primary osteoarthritic chondrocytes isolated from the femoral heads of OA-patients | Changes in HSPB6 protein level. |
| [34] | 50 Hz  0-100 µT  4 h | Human leukocytes | No effect on the gene expression level of *HSP*27, *HSP*70A or *HSP*70B. |
| [52] | 50 Hz,  1 mT, (SMF or PEMF)  30 min | *Escherichia coli* | Higher levels of DnaK and GroEL proteins induced by SMF. Lower levels induced by PEMF. |
| [37] | 50 Hz  1.0 mT  up to 2h | Mouse macrophages | Increased levels of HSP70 and HSP110 proteins at certain time points, but not generally. |
| [46] | 50 Hz  1 mT  3 h | K562 human leukemia cell line | Increase in HSP70 protein expression levels. |
| [50] | 60 Hz  8 µT ELF-EMF  1 h twice daily for 15 days post-transfection | The planarian *Dugesia dorotocethala* | Elevation in the level of HSP70 protein, activation of an ERK cascade, and increase in SRF-SRE binding. |
| [31] | 50 Hz  2 µT to 4 mT  15 to 30 min | HL-60, H9c2, and Girardi heart cells | No effect. |
| [35] | 60 Hz  8 and 80 μT  20 min | Human non-small cell lung cancer INER-37, and RMA E7 cell | Increased luciferase gene expression in INER-37 cells. No effect on RMA E7 cells. |
| [56] | 50 Hz  0-150 µT  30 min to 4 h | Transgenic strains of *C. elegans* | EMF strongly enhances the expression of the reporter gene lacz under the control of *HSP*16 or *HSP*70 promoters |
| [39] | Static MF  1 to 440 mT  16, 24, or 48 h starting at 24 and 48 h post transfection | Transfected rat primary cells | Increased (up to 3.5 fold) *HSP*70 reporter gene expression showed a dependence on flux density, exposure duration, and start time post transfection. |
| [49] | 50 Hz  300, 400, 600 µT  30; 2 x 30; 3 x 30 min | The bivalve *Mytilus galloprovincialis* | No effects at 300 µT.  At 400 and 600 µT exposed animals showed increase in HSP expression independent of exposure duration and concomitant with p38 MAP kinase signalling pathway activation. |
| [45] | 50-Hz  0.025-0.10 mT vertical or horizontal MF  1 h | Human leukaemia cell line K562 | Increase in HSP70, scavengers (melatonin or 1,10-phenantroline) inhibited the MF-induced increase in HSP70.at all flux density levels. |
| [32] | 60 Hz  6.3 or 8.0 µT  20 min | Human myeloid leukemia (HL-60) cells | No effect on HSP70 expression; small effect on HSF-HSE binding. |
| [53] | 5-100 Hz  14 mT rms for circularly polarized MFs and 10 mT rms for vertically polarized MFs | Escherichia coli K12 | No effect. |
| [33] | 50-60 Hz  100 µT  20 min to 24 h | Human keratinocytes | No effects in HSP27 as in the former study of HSP70 in breast or leukemia cells |
| [47] | 50 Hz  10-140 µT  30 min | Human myeloid leukemia (HL-60) cells | Induced HSP70 genes (A, B, and C) gene expression at all exposure levels peaking at 60-80 µT. |
| [40] | 15 Hz  2 mT  30 min | Cardiomyocytes isolated from neonatal Sprague-Dawley rats | Significantly increased HSP70 mRNA expression in hypoxic cardiomyocytes but MF exposure alone was not measured. |
| [57] | 50 Hz  3 mT | *D. melanogaster* | ELF-MF aggravated damages and enhanced thermal stress-induced HSP and OS response, no effect of MF alone. Exposure duration not provided. |
